# Data-Driven Object Pose Estimation in a Practical Bin-Picking Application

**DOI:** 10.3390/s21186093

**Published:** 2021-09-11

**Authors:** Viktor Kozák, Roman Sushkov, Miroslav Kulich, Libor Přeučil

**Affiliations:** 1 Czech Institute of Informatics, Robotics, and Cybernetics, Czech Technical University in Prague, Jugoslávských Partyzánů 1580/3, 160 00 Praha 6, Czech Republic; sushkov.r@gmail.com (R.S.); Miroslav.Kulich@cvut.cz (M.K.); Libor.Preucil@cvut.cz (L.P.); 2 Department of Cybernetics, Faculty of Electrical Engineering, Czech Technical University in Prague, Karlovo Náměstí 13, 121 35 Praha 2, Czech Republic

**Keywords:** random bin-picking, autonomous manipulation, CNN, industrial application

## Abstract

This paper addresses the problem of pose estimation from 2D images for textureless industrial metallic parts for a semistructured bin-picking task. The appearance of metallic reflective parts is highly dependent on the camera viewing direction, as well as the distribution of light on the object, making conventional vision-based methods unsuitable for the task. We propose a solution using direct light at a fixed position to the camera, mounted directly on the robot’s gripper, that allows us to take advantage of the reflective properties of the manipulated object. We propose a data-driven approach based on convolutional neural networks (CNN), without the need for a hard-coded geometry of the manipulated object. The solution was modified for an industrial application and extensively tested in a real factory. Our solution uses a cheap 2D camera and allows for a semi-automatic data-gathering process on-site.

## 1. Introduction

For several decades, production lines in various industries have relied heavily on the use of robotic manipulators. Traditional applications, such as welding automation, part manipulation, or assembly rely on precise predefined movements and defined positions of items in the robot’s workspace. However, recent advancements in robotic automation allow for the deployment of intelligent manipulators in tasks previously unsolvable by robots. The introduction of collaborative robots allowed robots to share the workspace with humans and perform cooperative tasks, while the advancements in machine learning in combination with fine force control in robotic manipulators enabled the automation of fine motor tasks, which could previously only be performed by a human operator. With the recent progress in deep learning, object pose detection intended for grasping and manipulation tasks has been intensely researched, inspiring research in various challenging applications. The increased affordability of robotic technology and the rising costs of human employees also represent incentives to increase automation in production.

Random bin-picking is a core problem in computer vision and robotics, where known objects are randomly placed in a bin and the task is to localize individual objects and determine the optimal gripping position for the manipulator. The problem has been a subject of intense research for several years and received support from various companies in the industry. One of the most prominent ones is the Amazon Picking Challenge [1], in which several research teams achieved outstanding results. However, the deployment of automated bin-picking applications on industrial production lines imposes strict requirements on reliability and robustness, making much of the existing work non-applicable to most real-world bin-picking problems due to their simplicity or lack of robustness for industrial use [2]. Current bin-picking methods often rely on expensive 3D cameras or the use of explicit CAD models of the manipulated parts, and the research on simple setups without the use of 3D vision systems or explicit models has been sparse until recent years, and practical applications in industry lines are rare.

### 1.1. Objectives and Contributions

Our paper addresses the bin-picking problem for industrial applications. We focus on specific metallic parts of a single type, which have to be picked from a transportation container. Metallic parts, often used in the automotive industry, are characteristic by their reflective surface, and their appearance is highly dependent on the light distribution, surface material, and camera viewing direction, which makes them unsuitable for traditional vision-based approaches [3]. We propose a solution using a simple 2D camera and a direct light source, both mounted directly on the robot’s gripper, which allows us to take advantage of the reflective properties of the part. Optimal gripping positions of individual parts are estimated with a two-phase object detection and localization pipeline, using feature descriptors for part detection and a CNN neural network trained for position and orientation estimation.

The developed vision-based localization system is the main contribution of this work. It takes full advantage of the reflective properties of metallic parts, allows for the use of a cheap 2D camera, and is computationally inexpensive, making it a viable low-cost solution for industrial use. The developed pose estimation method was also tested on both synthetic and real-world data. A semi-automatic data collection process allowing on-site data acquisition was developed as a part of the system. We have also created a new publicly available dataset (http://imr.ciirc.cvut.cz/Downloads/Datasets, accessed on 9 September 2021) with labeled images containing reflective metallic parts. Finally, the developed bin-picking solution was modified for industrial use and was successfully deployed and extensively tested in factory production.

### 1.2. Paper Outline

The rest of this section is dedicated to related work. Section 2 presents the proposed bin-picking solution and system specifications. The vision-based object detection and localization method is described in detail in Section 3. The performance of the system and experimental results are shown in Section 4. Finally, conclusions and final remarks are given in Section 5.

### 1.3. Related Work

In random bin-picking, object positions or optimal grasping positions are typically estimated directly from 2D or 3D images. While 3D vision provides additional depth information, industrial 3D cameras are still rather expensive, and although low-cost sensors, such as Microsoft Kinect or RealSense, provide 3D data of similar quality, such sensors are unsuitable for use in industrial environments. Moreover, the data representation itself is more complicated and the computation and memory requirements are higher, as an extra dimension is added [4]. Lastly, 3D vision systems have lower resolution and suffer from noise and artifacts, making the depth estimate insufficient or unreliable for some applications, where this is especially notable in scenes containing light reflections [5].

Several methods for object pose estimation using 2D camera images were introduced over the years. Sparse 2D–3D feature matching is a popular approach to pose estimation, in which visual features are matched between an input 2D image and a 3D object model [6]. However, the large changes in appearance of reflective objects make it intractable to find corresponding visual features between views of the same object from distinct positions. Additionally, this approach is not well-suited for textureless objects [7]. It is also difficult to construct precise 3D models without expert knowledge or specialized scanning devices [8]. Other conventional approaches rely on template matching, where the object pose is estimated by correlating the input image with image templates from a reference database [9,10]. While these approaches are suitable for textureless objects, reflective objects are highly view- and lighting-dependent, and a large number of templates would be necessary to cover all variations to guarantee detection across all positions. An interesting method suitable for reflective textureless objects was presented in [3] and further extended in [11]. The method is motivated by photometric stereo and uses an imaging system based on multiple light sources to acquire a multi-light image with individual channels (red, green, and blue) from different illumination directions. Similarly to our proposed system, this method takes advantage of the reflective properties of the object; however, the lighting setup presented in [3,11] is significantly more complicated and limiting, and the method depends on a CAD model of the localized object.

Recent advancements in deep learning resulted in major breakthroughs in image understanding as new techniques for object detection and localization became available [12]. As a result, research on simple 2D image setups for bin-picking, without the use of 3D vision or explicit CAD models, has once again become the center of attention. Various methods for six degrees of freedom (6DoF) object pose estimation based on the use of Convolutional Neural Networks (CNN) are drawn to attention. Some recent methods use CNNs to regress 2D keypoints, using them as an intermediate representation for the Perspective-n-Point (PnP) algorithm to compute 6DoF pose parameters [13,14,15], and other methods improve on this approach by using pixel-wise predictions to provide a flexible representation for localizing occluded or truncated keypoints [8,16].

A different approach is used by methods based on end-to-end neural networks. Grasp learning in an end-to-end manner, without an intermediate representation of the object pose, is conceptually elegant, since end-to-end methods take an image as input, and the output is a corresponding pose. Such methods estimate the grasping position either directly [17] or in combination with other approaches [18,19,20]. However, generalization is an issue here, since these methods suffer from high demand for labeled data for training, and data acquisition is usually time-consuming and laborious. Multiple robotic manipulators can be used for learning, but such approaches highly increase the cost of the equipment [21,22].

Several large-scale datasets were introduced, often associated with both real and synthetic RGB and RGB-D data, and enhanced by a database with object models for easier incorporation in manipulation and planning software platforms [23,24]. Notable is the latest BOP benchmark dataset [25], which relates to the BOP Challenge 2020 [26], where state-of-the-art methods are compared in various categories on a large collection of datasets. Benchmark datasets can be used for method verification and qualitative comparison of developed methods; however, real-world applications for new objects still require the acquisition of training data or a 3D model of the object of interest.

## 2. System Specification

**Figure 1:**
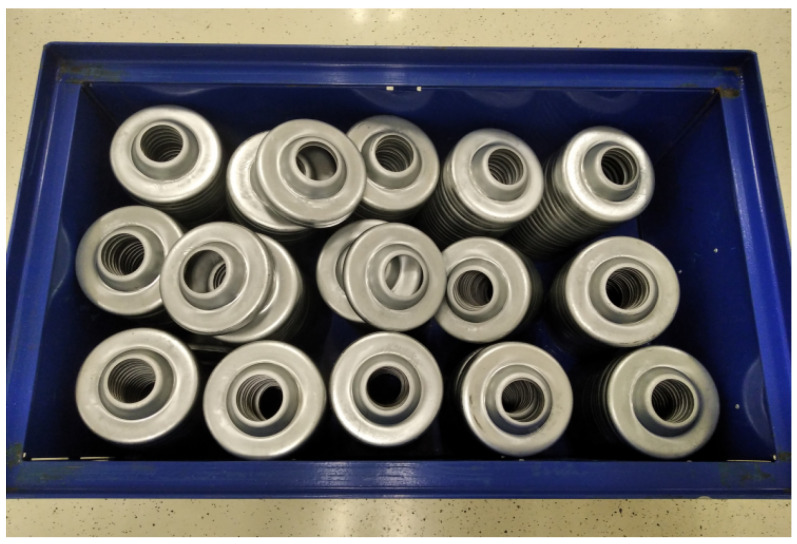
Distribution of parts in the transportation container.

The developed solution addresses a practical pose estimation task motivated by a real-world problem of random bin-picking of textureless metallic automotive parts requested for automation. The task is to localize and secure metallic strut brackets from a transportation container and feed them to an automated assembly line. The parts are semi-randomly distributed in a transportation container, as can be seen in Figure 1. Due to their shape and the way they are initially placed in the transportation container, the parts are generally facing upwards, with a maximal tilt of 25 degrees. This allows for easier problem definition. In regard to this distribution, the gripper was designed to pick the parts from the top. The parts are originally organized in columns, but get scattered during transport, which may result in occasional occlusions. An occlusion detection module was developed and added into the object localization pipeline, in order to securely determine the topmost part.

The developed robotic cell should be robust for use in a factory and must account for external factors from the environment, such as occasional disruptions in the workspace or changes in lighting conditions. Since the workspace will be located in an open area with occasional passersby, a collaborative UR10 robot was selected for the task. The final version of the developed robotic workcell is described in detail in the factory setup Section 4.2.

**Figure 2:**
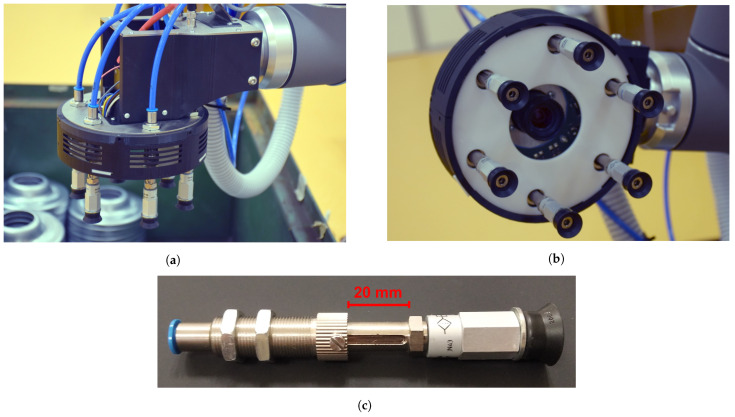
Robotic gripper with suction cups, an integrated lighting system, and a 2D camera positioned at its center. (**a**) Side view. (**b**) Bottom view. (**c**) A detailed view of a suction cup with the integrated spring mechanism.

The robot was equipped with a robotic gripper of our own design. The gripper is equipped with six vacuum suction cups with integrated springs which allow for a 20 mm compression (Figure 2c), a 2D camera mounted at the center of the robotic gripper for accurate pose estimation, and an integrated lighting system. The lighting system consists of an annulus circuit board with LED lights integrated inside the gripper and a white diffuser plate overlapping it from the outside, as can be seen in Figure 2b. This light configuration is used to provide direct light from a constant position relative to the camera. We use a Smartek GC2591MP camera (FRAMOS GmbH, Taufkirchen, Germany) with a CMOS MT9P031 1/2.5 sensor (ON Semiconductor, Phoenix, AZ, USA) and a resolution of 2592 × 1944 pixels. The lens used in this setup was a Computar M0814MP2 (CBC AMERICA, Cary, NC, USA) with a focal length of 8 mm and an angle of view of 67${}^{\circ }$. The developed laboratory prototype can be seen in Figure 2.

We propose an object localization pipeline based on a combination of feature descriptor-based image segmentation and the use of an end-to-end trained convolution neural network (CNN) for pose estimation. The method is fully data-driven, that is, the localization pipeline is trained using a collection of data (labeled images), without the need for a model of the object. Most data-driven approaches need a large amount of labeled data, therefore a semi-automatic training process was proposed and developed to allow for an easy transition to different robotic cells and manipulated objects.

### 2.1. Control System

**Figure 3:**
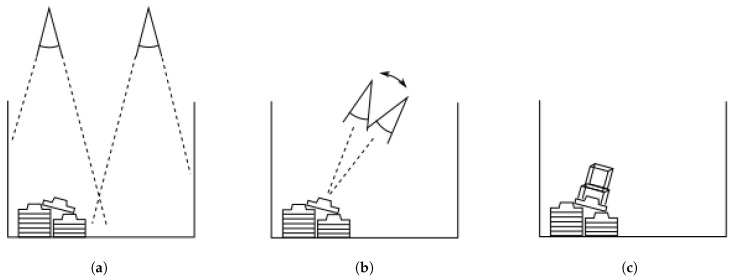
Control algorithm phases. (**a**) Selection. (**b**) Iterative alignment. (**c**) Picking.

The main objective of the application is to detect, localize, and acquire parts from the transportation container. This section describes the implementation of the control algorithm for bin-picking used in the developed framework. The main control loop is schematically shown in Figure 3 and composes of three main operations: selection, alignment, and picking.

The bin-picking process begins with the selection phase. Due to the limited field of view of the camera and robot’s operation space, it is necessary to capture several images in fixed positions above the transportation container to cover the whole area (our setup requires three images for the selection phase). The initial detection is performed using these images and a list of detected object candidates and their estimated positions is generated. Additional filtering is performed during each object detection, wherein occluded objects are removed from the list and the remaining candidates are sorted according to their estimated positions, from the highest placed part to the lowest.

The control loop iterates through the list of detected parts. The robot moves directly above the selected part and captures the image of the part once again, starting the alignment phase of the process (shown in Algorithm 1). During this phase, the gripper is iteratively aligned with the normal vector of the part in an optimal position. A new pose is estimated during each iteration, and the camera is moved to the position where its *z*-axis matches the estimated normal vector at a constant distance (δ=0.25 m) from the estimated part origin. The iterative alignment is applied in order to minimize the position estimation error. In the following text, we will be referring to this method as iterative close-up.

The acquired object position is then used in the last phase of the process, described in Algorithm 2. The gripper is moved a set distance (β=0.05 m) above the estimated position, and an attempt is made to acquire the part. At this point in the process, the motion control is switched to a force-sensitive mode in which the performed motion is stopped when the overall external force applied to the gripper exceeds a set threshold. The force feedback provided by the UR10 robot is sensitive enough to detect a compression of the suction cup spring mechanism from Figure 2; therefore, no additional force-torque sensors were required. The 20 mm compression interval has allowed us to experimentally estimate a safe force threshold used to stop the motion of the robot when the springs are sufficiently compressed. After the motion is stopped, the suction mechanism is activated in an attempt to grip the part and the griper is elevated. The control program evaluates whether the grip was successful by measuring the pressure on individual suction cups. Successfully picked parts are then placed into the assembly line.
**Algorithm 1** Alignment loop
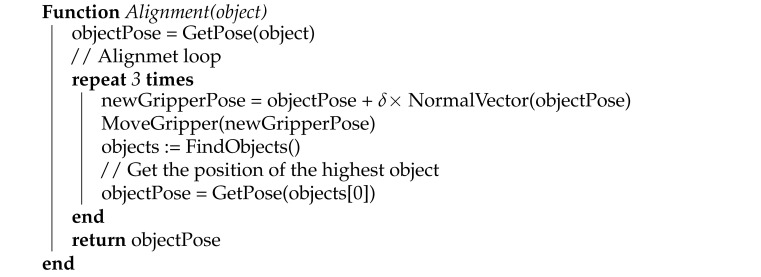

**Algorithm 2** Picking up the object
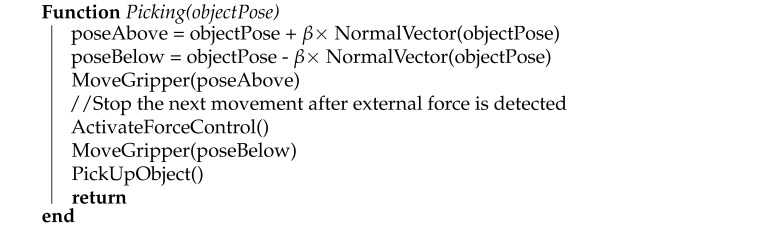



### 2.2. Camera and Lighting System

**Figure 4:**
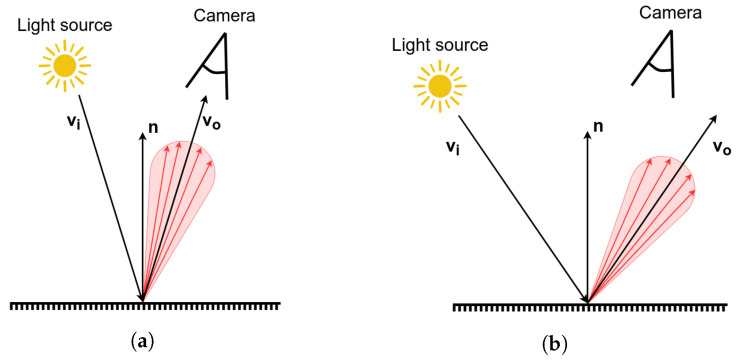
Change in light on a highly specular surface. (**a**) Direct reflection to the camera. (**b**) Reflection in a different direction.

The visual appearance of textureless objects is dominated by their global shape, color, reflectance properties, and the configuration of light sources (Figure 4). As such, vision-based methods are often highly susceptible to changes in lighting conditions when applied to textureless objects, and many traditional recognition methods relying on photometric local patch detectors often fail to reliably recognize and locate the objects [27].

**Figure 5:**
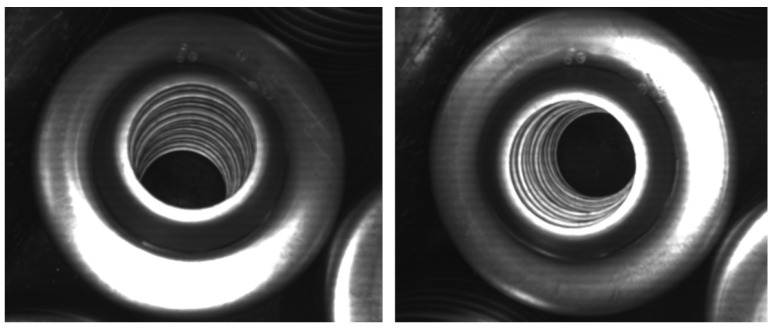
Comparison of two images of the manipulated part captured from different angles.

The developed system uses a light source installed directly in the robotic gripper, together with a 2D camera used for object localization. By using a pre-set high-intensity light from close proximity, we can limit the influence of light from the surrounding environment. Taking images from close proximity to the object also improves the accuracy of the prediction and allows for the use of a relatively cheap 2D camera. Many industrial parts are metallic and their surfaces are highly reflective. As stated above, the appearance of these reflective parts is highly dependent on the camera viewing direction, as well as the distribution of light and its position relative to the object, and the same behavior can be seen in the manipulated parts in this application (Figure 5). The idea of a light in a fixed, known position to the camera allows us to use these properties as additional features used to determine the position and orientation of the object.

### 2.3. Semi-Automatic Data Collection

Data-driven approaches often need large quantities of labeled data. Getting labeled images from a variety of poses is usually time-consuming and often a manual task, since some approaches need over 10,000, or even 100,000 labels [21,22].

The proposed system allows for a semi-automatic data collection without the need for any special constructions or conditions and can, therefore, be used to train the pose estimation system on-site, making the system easily deployable and further improving the robustness of the system. We start by manually placing the end effector of the robot on the manipulated object in an optimal gripping position. This ground-truth position is later used while labeling the data for supervised learning.

**Figure 6:**
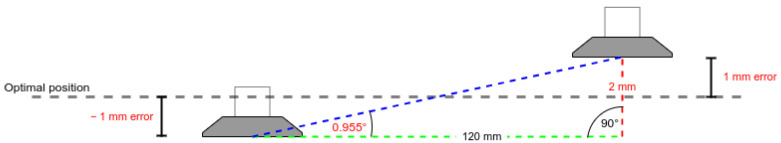
An illustration of the maximal depth and orientation errors.

The UR10 robot enables a freedrive mode in which the operator can manually guide the end effector by hand. This allows for easy and precise manipulation. Thus, the achieved precision is almost entirely dependent on the ability of the operator to recognize and achieve the optimal position. We are strongly confident that we are able to discern any displacements over 1 mm in both the horizontal and vertical directions. Thus, we estimate the maximal displacement error along the *x*, *y* and *z* axes as ±1 mm. The maximal orientation error can be derived from the *z* error (Figure 6). The distance between two opposing suction cups is 120 mm and the maximal possible displacement is 1 mm on one suction cup and −1 mm on the other. Using basic trigonometry, we can determine the maximal orientation error to be ±0.955${}^{\circ }$. Although suboptimal, the resulting precision is sufficient for the intended application. Moreover, it allows us to acquire the training images in a way that is identical to the acquisition during actual operation without reliance on fixed object holders, markers, or synthetic data.

After the initial step, we start the process of automatic data collection. The robot moves above the object and collects images of the object from randomly generated positions at different distances and angles within predefined boundaries. As many as 50 to 200 images are collected for each initial position.

As will be described in the following paragraphs, CNN training and object localization are performed using rectangular image patches generated by a bounding box detector. However, the object only covers approximately 70% of the image patch, and the rest of the patch contains neighboring parts or the transportation container. It is necessary to repeat the data acquisition procedure with the parts placed in different positions in the container to prevent the CNN from learning a set background pattern. This is similar to the process used in many frameworks, where synthetic object images are generated with different backgrounds. This is especially important for metallic parts and other objects with reflective properties.

Using the original ground-truth positions and the positions of the robot at which the images were captured, a relative position between the camera and the part is computed for each image. These positions are used as labels in the training and testing data. Creating the training data with the use of the relative position of the camera to the object allows us to easily transfer the solution to a different robot or application.

## 3. Object Detection and Localization

**Figure 7:**
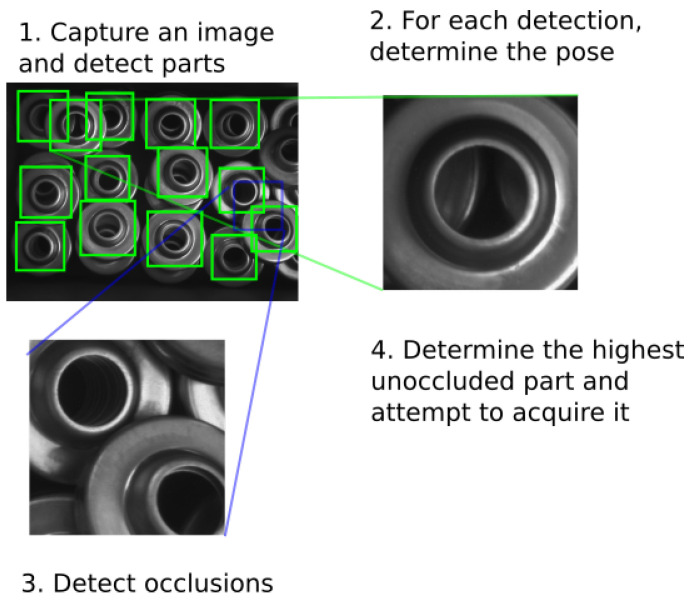
The developed framework consists of a part detector, a pose estimator, and an occlusion detector.

The pose estimation process can be divided into three main parts (Figure 7). The first part is the object detection described in Section 3.1, where image patches containing the detected objects are selected from the image. The second part uses an end-to-end trained CNN-based neural network to determine the position and orientation of the object from the image patch. In Section 3.2, we define the position and orientation parameters, and Section 3.2.3 is dedicated to the end-to-end neural network. The final part determines possible occlusions with neighboring objects, and is described in Section 3.3. Using the previous steps, the highest unoccluded object is then selected for manipulation.

Since the application is focused on textureless metallic parts, we have to consider their characteristics in the image processing phase. To compensate for some of their properties, we have decided on the use of simple grayscale images instead of RGB images. Since the parts have no colorful texture, there is no significant loss in information, and their reflective properties may cause additional undesirable color information from the neighboring environment to be present in the image, possibly affecting the function of the vision system. Furthermore, using only a simple grayscale image speeds up the image processing system.

### 3.1. Image Segmentation

Histogram of oriented gradients (HOG) [28] was used for the first part of the pose estimation process. HOG is a feature descriptor often used for object detection, it is easy to train, and it often performs well for a range of different problems. While the recent development in deep learning has resulted in various new methods that outperform traditional computer vision methods in several aspects, the use of deep learning often introduces additional difficulties in training and deployment, common downsides being the higher requirement on the number of labeled training data, and a larger model size [29,30]. The use of deep learning object detection methods (such as YOLO [31] or Faster R-CNN [32]) can significantly increase the speed of object detection; however, this is often contingent on the use of expensive hardware with high computational power, the use of which might be cost-ineffective in industrial applications.

**Figure 8:**
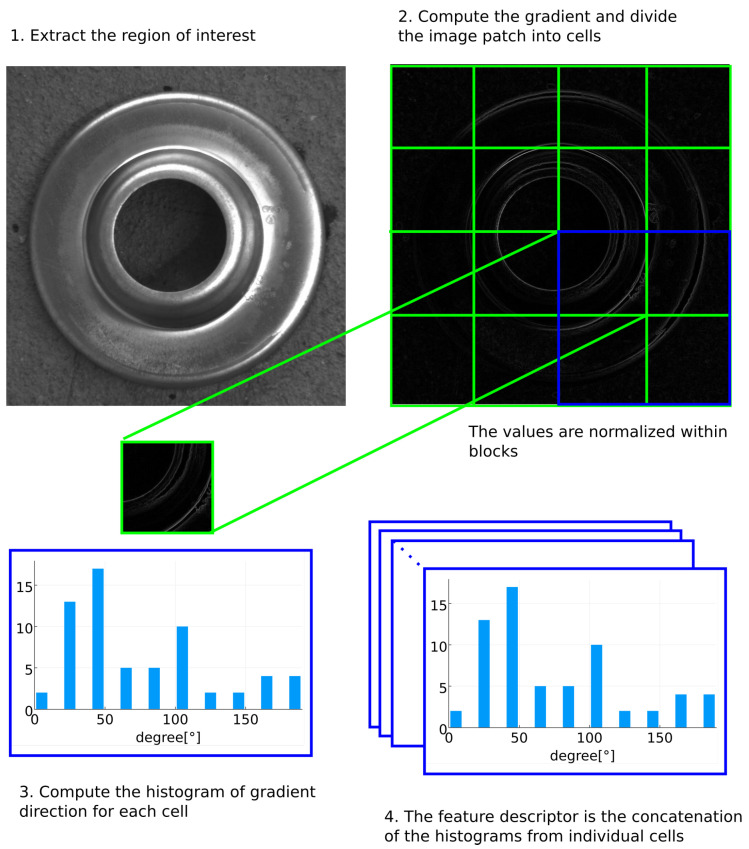
Histogram of oriented gradients.

In this method, the image is divided into small adjacent cells (e.g., of size 8 × 8 pixels), and each cell is represented by its histogram of gradient directions. The cells are further combined into larger image blocks to improve the accuracy of the method. A feature vector for each region of interest is then formed by the concatenation of individual cell histograms (Figure 8). The sliding window approach [33] is used, allowing us to use HOG to detect and localize objects in the image. Using this approach, a window is moved across different regions of the image and for each region, a feature vector is determined. A linear SVM [34] classifier is then used to classify each feature vector. The detector has been trained on a training set containing 1058 training images. From the training set, only 53 were positive samples containing the desired part, and 1005 were negative samples generated from random images. The parameters used for the HOG detector are: block size 16 × 16, cell size 8 × 8, and image patch size 64 × 64.

The output of the object detection is a set of bounding boxes with detected positions of objects in the image, defined by their width, height, and their original position in pixel coordinates. Since the object is circular, the detected area should generally be rectangular. The detection algorithm was limited to provide only rectangular bounding boxes, practically reducing the bounding box representation only to its origin coordinates and width, all provided in pixels (Equation (1)). Henceforth, we will be establishing a naming system in which all pixel coordinates measured in the image plane will be marked with a superscript *p*, such as: $x^{p}$. The bounding box parameters and image patches defined by them are then used for exact pose estimation in the second phase of the localization pipeline.

(1) $$b_{box}=\left[\begin{array}{ccc} x_{b}^{p} & y_{b}^{p} & w_{b}^{p} \end{array}\right]$$

Since the design of the two-phase localization pipeline is modular, it is possible to choose a suitable object detector at one’s discretion, as long as the training data for the end-to-end network are generated by the same object detector that is used in the application. The HOG algorithm was chosen in the initial phase of the project due to its simplicity, speed, and low requirement for labeled training data, and was used for the full duration of the project.

### 3.2. Pose Estimation

**Figure 9:**
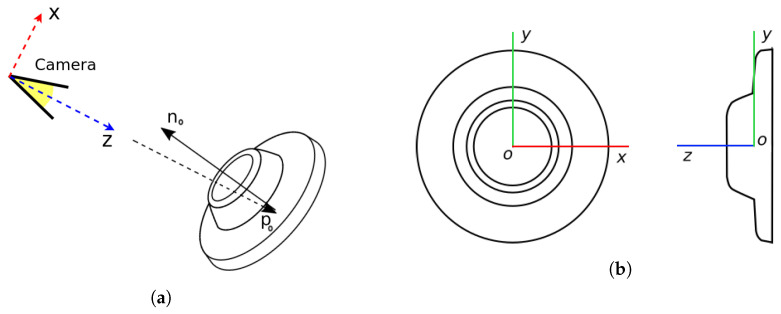
The part coordinate system. (**a**) Position and orientation of the part. (**b**) Object coordinate system.

Precise estimation of the position and orientation of the object is necessary for the picking task in an unstructured environment. Incorrect alignment with the object during contact with the gripper may lead to an unsuccessful grip or even damage to the gripper or other robot parts, which may be difficult or expensive to repair. Object pose is composed of the position and the orientation of the part relative to the camera or another coordinate system, as shown in Figure 9a. Vector $p_{o}$ represents the position of the object origin relative to the camera and the part normal $n_{o}$ determines the relative orientation of the part. The camera coordinate system used in this paper follows the standard computer vision convention, where the *z* axis is orthogonal to the image plane. Due to the design of the lighting system, we can consider the light source to be at the same position as the camera.

(2) $$\begin{array}{c} p_{o}=\left[\begin{array}{c} x_{o}\\ y_{o}\\ z_{o} \end{array}\right],\;n_{o}=\left[\begin{array}{c} n_{xo}\\ n_{yo}\\ n_{zo} \end{array}\right],\;∥n_{o}∥=1 \end{array}$$

The large, flat disc surface on the top of the object is used as a contact surface for grasping by the suction cups. The part coordinate system is shown in Figure 9b. The origin of the coordinate system coincides with the center of the part, and the *z*-axis is defined by the part normal. The orientation of the *x* axis can be selected arbitrarily since the part is rotationally invariant. From this definition, we can see that this part has five degrees of freedom, and that infinitely many coordinate systems may be assigned to it. Despite this ambiguity, this formulation is useful, and it is used in this framework for pose determination.

For the task of precise pose estimation, we propose an end-to-end neural network based on regression. The input of the network is the image patch *X* defined by a bounding box from the object detection phase. The output is a vector of position coefficients $\hat{y}$ that determine the object position. The training data are generated by capturing an image of a part from a random position. Both the part and camera positions are known and are in the base coordinate system of the robot. To train the model efficiently, it is necessary to express the part pose in the camera coordinate system. In the following sections, we derive the pose in a form suitable for regression training. We describe the neural network in depth in Section 3.2.3.

#### 3.2.1. Object Normal Vector

We mark the homogeneous coordinates of the object normal expressed in the object coordinate system as ${\bar{p}}_{zo}$, and the coordinates of the object origin as ${\bar{p}}_{0o}$.

(3) $$\begin{array}{c} {\bar{p}}_{zo}=\left[\begin{array}{c} 0\\ 0\\ 1\\ 1 \end{array}\right],\;{\bar{p}}_{0o}=\left[\begin{array}{c} 0\\ 0\\ 0\\ 1 \end{array}\right] \end{array}$$

These vectors can be expressed in the gripper coordinate system as:

(4) $${\bar{p}}_{zg}=T_{go}{\bar{p}}_{zo}\;,\;{\bar{p}}_{0g}=T_{go}{\bar{p}}_{0o}\;,$$

where $T_{go}$ is the transformation matrix from the gripper to the object. The object normal expressed in the gripper coordinate system is the difference between these two vectors in non-homogenous coordinates:

(5) $$n_{o}=p_{zg}-p_{0g}\;,$$

where $p_{zg}$ and $p_{0g}$ are non-homogeneous versions of ${\bar{p}}_{zg}$ and ${\bar{p}}_{0g}$. Equation (5) is used to determine the value of the normal vector $n_{o}$ from the pose of the camera and the pose of the object.

#### 3.2.2. Object Position

The output value $\hat{y}$ of the neural network is the value of the normal vector followed by *position coefficient* parameters, which are used to determine the position of the object relative to the camera coordinate system. The model is built in such a way that it predicts the normal vector directly. However, the same approach cannot be used for a direct prediction of the part position, since the image patch used as an input for the neural network does not contain any information about the position and size of the bounding box, from which it was generated. A possible way around this problem would be to use the information about the size and position directly in the neural network. However, this would complicate the structure of the network, since it would require two entry points, the first for the image patch and the second for the image patch parameters.

Our solution to overcome this problem is to avoid predicting the distances directly, and instead of that, use the image patch position and width for first-order estimates, and train the neural network to predict coefficients that improve the initial accuracy. The position values predicted by the neural network are position coefficients $p_{c}$, which we define in the following paragraphs.

(6) $$\begin{array}{c} p_{c}=\left[\begin{array}{c} x_{bo}^{p}\\ y_{bo}^{p}\\ k_{d} \end{array}\right] \end{array}$$

These are used to determine the position of the object relative to the camera coordinate system. The predicted coefficients $x_{bo}^{p}$, $y_{bo}^{p}$ and $k_{d}$, and the original bounding box parameters (Equation (1)) are used to determine the distance from the camera to the object and the translation of the camera in the direction of the *x* and *y* axes.

**Figure 10:**
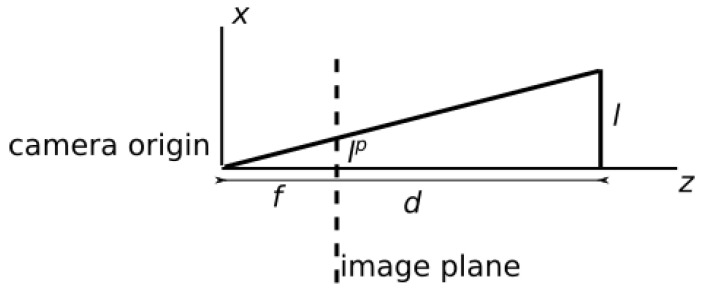
Pinhole camera model.

To describe this functionality, we need to consider a projection of an object on the image plane. Consider a simple pinhole camera model (Figure 10). An object represented with world coordinates *l* and *d* is present. The object is projected onto the image plane at a coordinate $l^{p}$. The focal length *f* determines the distance from the origin of the camera coordinate system to the image plane. The focal length of a camera can be determined through intrinsic calibration. Assuming that the focal length is known, the projection can be determined with Equation (7). The image coordinates of the object can be transformed into world coordinates and used to calculate the part position and the distance to the object.

(7) $$l^{p}=\frac{lf}{d}$$

##### Distance Prediction

We base the model for predicting the distance from the camera to the object on the following equation:

(8) $$d=\frac{wf}{w^{p}}\;,$$

where *d* is the distance, *w* is the diameter of the object, *f* is the focal length of the camera, and $w^{p}$ is the diameter of the object measured in pixels. The general idea of this approach is to predict the size of the object in pixels and use this equation to determine the camera-object distance. However, in order to use this equation, it is necessary to know the size of the object. Although it is possible to provide measurements of the object, it is more convenient to derive a more general formula and to avoid using concrete values when possible. Let us simplify Equation (8). First, $w^{p}$ can be expressed as

(9) $$w^{p}=\alpha w_{b}^{p}\;,$$

where $w_{b}^{p}$ is the width of the image patch from the original bounding box parameters (Equation (1)). We introduce the coefficient α whose value represents the ratio between the bounding box width and the real diameter of the object in pixels. It is different for every image patch, and the value is usually close to 1 because the width of the image patch is approximately equal to the size of the object. However, due to the unstable visual characteristics of reflective objects, the precision of the bounding box estimation was often suboptimal, and further refinement was needed. This yields:

(10) $$d=\frac{wf}{\alpha w_{b}^{p}}$$

Now, we substitute

(11) $$k_{d}=\frac{wf}{\alpha }$$

from which we get the following equation:

(12) $$d=\frac{k_{d}}{w_{b}^{p}}$$

The difference between Equations (8) and (12) is that we do not need to know the specific parameters of the object and camera *w* and *f*. By training the model to predict $k_{d}$, we can use it to determine the distance to the object. It should be noted that the focal length *f* is used later for the estimation of the $x_{bo}^{p}$ and $x_{bo}^{p}$ coefficients (Equation (17)).

##### Prediction of Translation in *xy* Plane

**Figure 11:**
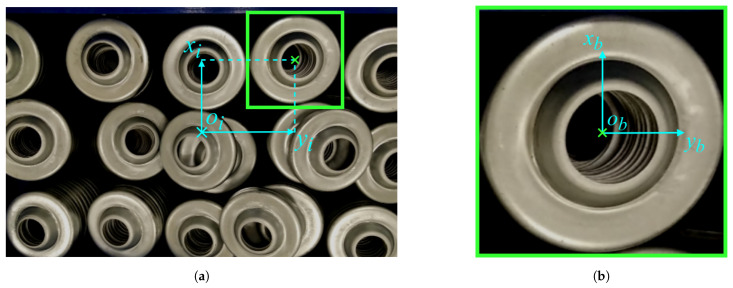
Translation prediction in the *xy* plane. (**a**) Image coordinates. (**b**) Bounding box coordinates.

The prediction model for the translation of the camera in the direction of the *x* and *y* axes is illustrated in Figure 11. To determine the position, two coordinate systems are established. The image coordinate system with the origin in the center of the image and axes as shown in Figure 11a, and the bounding box coordinate system with the origin in the center of the bounding box surrounding the object shown in Figure 11b. Since multiple coordinate systems are used in this section, it is necessary to establish a naming convention for the coordinates. The superscript determines whether the coordinate is measured in space (*x*) or in the image plane ($x^{p}$); the first subscript determines the coordinate system: $x_{i}$ is the camera (image) coordinate system, $x_{b}$ is the bounding box coordinate system; the second subscript is used to select the item whose coordinates are measured (whether coordinates of the object $x_{io}$ or the bounding box $x_{ib}$).

The object position coordinates $x_{io}$ and $y_{io}$ are determined using the following equations:

(13) $$\begin{array}{c} x_{io}=\frac{x_{io}^{p}d}{f}\;,\;y_{io}=\frac{y_{io}^{p}d}{f} \end{array}$$

where *d* is the distance from the camera to the object, *f* is the focal length of the camera, and $x_{io}^{p}$ and $y_{io}^{p}$ represent the object coordinates in the image plane. The transformation between the image and bounding box coordinate systems is performed using the following equations:

(14) $$\begin{array}{c} x_{io}^{p}=x_{ib}^{p}+x_{bo}^{p}\;,\\ y_{io}^{p}=y_{ib}^{p}+y_{bo}^{p} \end{array}$$

where $x_{ib}^{p}$ and $y_{ib}^{p}$ mark the position of the bounding box center, and are calculated from the bounding box parameters from Equation (1). The position coefficients $x_{bo}^{p}$ and $y_{bo}^{p}$ represent the correction shift between the center of the detected bounding box and the exact center of the object in the image plane. Since the other parameters are known from the object detection phase, the model is trained to predict the position coefficients $x_{bo}^{p}$ and $y_{bo}^{p}$ in order to determine the exact position of the object.

After determining the pixel coordinates in the image coordinate system, we obtain the $x_{io}$ and $y_{io}$ coordinates in the camera coordinate system by applying Equations (13) and (14). The coordinates of the object can be expressed as:

(15) $$\begin{array}{c} x_{io}=\frac{x_{io}^{p}d}{f}=\frac{x_{ib}^{p}+x_{bo}^{p}}{f}d\;,\\ y_{io}=\frac{y_{io}^{p}d}{f}=\frac{y_{ib}^{p}+y_{bo}^{p}}{f}d \end{array}.$$

#### 3.2.3. End-to-End Neural Network

We propose a regression model based on CNN for the task of part pose estimation. The network is based on the TensorFlow [35] open-source library for machine learning developed by Google. TensorFlow provides efficient components and a high-level interface for building, training, and running neural networks and other machine learning models. It supports different hardware for training and running the models, and is cross-platform.

**Figure 12:**
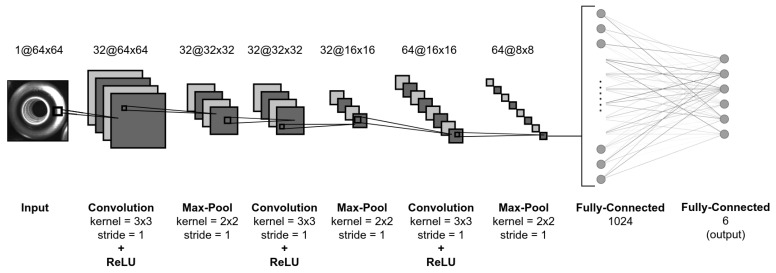
CNN architecture for object pose estimation.

After identifying the regions of the captured image that contain objects, image patches are extracted for pose estimation. These are resized to 64 × 64 pixels and used as an input for the network. This is followed by three convolutional layers with the ReLU activation function, followed by max pool layers. A large number of max pool layers is used to minimize the number of parameters of the model, and this is needed because of the sparsity of data. The structure of the network is shown in Figure 12. The last layer of the model network is a fully connected layer with six neurons. The output $\hat{y}$ is the predicted value of the object normal $n_{o}$ (Equation (2)) and position coefficient parameters $p_{c}$ (Equations (6) and (17)).

(16) $$\begin{array}{c} \hat{y}=\left[\begin{array}{c} n_{o}\\ p_{c} \end{array}\right] \end{array}$$

The size of the input (64 × 64) has been selected as a trade-off between the level of detail of the image and the number of parameters of the network. Having a larger input would capture the object with more detail, but it would also increase the total number of weights of the network, which would increase the computational complexity and the size of the model.

The CNN model is trained in a supervised learning fashion. The training data consist of a set of pairs (*X*, *Y*), where *X* is the input of the network (an image patch of the detected object) and *Y* is the output (part normal and position coefficients). In order to generate the training data, the position coefficients need to be determined. Assuming that the camera pose and the object pose are known, the position coefficients are obtained as follows:

(17) $$\begin{array}{c} k_{d}=dw_{b}^{p}\;,\\ x_{bo}^{p}=\frac{x_{io}f}{d}-x_{ib}^{p}\;,\\ y_{bo}^{p}=\frac{y_{io}f}{d}-y_{ib}^{p} \end{array}$$

The full output vector *Y* is:

(18) $$\begin{array}{c} Y=\left[\begin{array}{c} n_{xo}\\ n_{yo}\\ n_{zo}\\ x_{bo}^{p}\\ y_{bo}^{p}\\ k_{d} \end{array}\right],\;n_{o}=\left[\begin{array}{c} n_{xo}\\ n_{yo}\\ n_{zo} \end{array}\right],\;p_{c}=\left[\begin{array}{c} x_{bo}^{p}\\ y_{bo}^{p}\\ k_{d} \end{array}\right] \end{array}$$

An Adam optimizer with a learning rate of 0.001 was used, 250,000 training iterations were required, and a dropout rate of 0.5 was used for regularization. The loss function used for optimization is the mean squared error function, which is suitable for the regression task. The function is shown in Equation (19), where $\hat{y}$ is the predicted value and *y* is the ground truth. In practice, neural networks are often trained in mini-batches, which means that *N* different inputs are presented to the network, and the loss is computed as the average of individual errors. We used the mini-batch size of 30. The equation for average loss is shown in Equation (20).

(19) $$V\left(\hat{y},y\right)={\left(y_{i}-{\hat{y}}_{i}\right)}^{2}$$

(20) $$L=\frac{1}{N}\sum_{i=1}^{N}V\left(\hat{y},y\right)$$

### 3.3. Occlusion Detection

If the objects are randomly placed inside the bin, an occlusion may occur, making some of the objects temporarily unavailable for picking. The occluded objects need to be removed from the list of candidates to maximize the picking success rate and to avoid damaging parts of the robot or the gripper.

**Figure 13:**
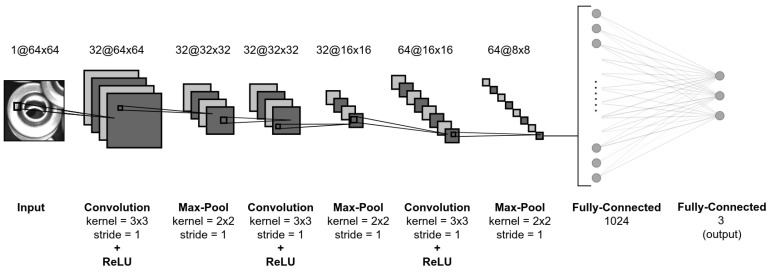
CNN architecture for occlusion detection.

In order to determine whether a part is occluded, we enlarged the original bounding box by 50% to encompass its larger neighborhood and provide additional information about its surroundings. The generated image patch is resized to 64 × 64 pixels and used as an input to a convolutional neural network trained as an occlusion classifier. The network is structured in a similar way as in Section 3.2.3, using three convolutional layers with ReLu activation function, followed by max pool layers. However, the last fully-connected layer contains only three neurons, serving as the output of the occlusion classifier. The structure of the network is shown in Figure 13.

**Figure 14:**
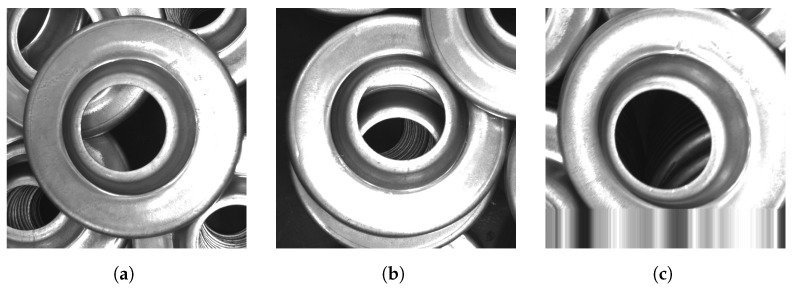
Example cases for part occlusion. (**a**) Unoccluded part. (**b**) Occluded part. (**c**) Unclear occlusion information.

The three possible outputs of this model are ‘occluded’, ‘unoccluded’, and ‘unclear’. An example for each of these cases is provided in Figure 14. While the first two outputs are self-explanatory, the last ‘unclear’ state was included in the model to compensate for cases where the image was corrupted or where the object is not fully captured in the image, in which case the enlargement of the original bounding box resulted in the input image patch partially extending out of the image. The output of the fully-connected layer corresponds to one of the possible classifications, and the neuron with the highest value represents the final prediction of the model.

Training data for this model was labeled manually. We used approximately 100 training images, and the dataset was augmented eightfold by (a) rotating the image 90, 180, and 270 degrees, (b) flipping the image upside down, and (c) combining steps a and b. Adam optimizer with learning rate 0.001 and 250,000 training iterations was used for training. The dropout rate was set to 0.5. The loss function used for training was the cross-entropy loss shown in Equation (21), which is often used for classification tasks. The batch loss was defined in the same way as in Equation (20).

(21) $$V\left(\hat{y},y\right)=-y\ln\left(\hat{y}\right)-\left(1-y\right)\ln\left(1-\hat{y}\right)$$

## 4. Results

The system was originally developed in a laboratory environment using a Kuka KR 5 arc robot and later modified to use the UR10 collaborative robot, the reason being the intended workspace in an open area with possible passersby. The laboratory setup was used to develop and fine-tune the necessary algorithms and gather initial training and testing data. The final system was then moved to the factory and deployed in production.

### 4.1. Accuracy and Speed

**Figure 15:**
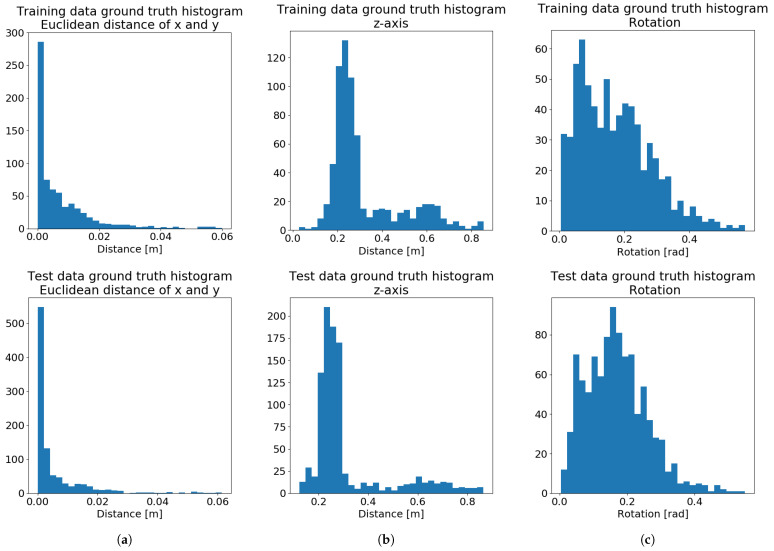
Training (top) and test (bottom) data distribution depicting: (**a**) Deviations in the *x* and *y* axes. (**b**) The distance from the object. (**c**) Orientation deviations.

We gathered close to 3000 labeled images during the initial phase of the deployment in the factory. Approximately 70% of these images were used for the development, training, and optimization of neural networks for pose estimation. The other 30% were used to test the trained networks. Position distribution of the testing data are depicted in Figure 15. To achieve high accuracy, most of the images were taken from a close proximity of the expected work distance used in the iterative close-up algorithm. However, to guarantee the robustness of the trained networks, a certain number of the gathered images varies in distances and angles from which they were captured, as well as the relative position of the object in relation to the center of the image.

Various training data distributions were tested to optimize the performance of the localization system. We designated approximately 2000 images for the training of neural networks for pose estimation. However, the number of training images can be reduced by delicate data selection, which would still cover the expected range of positions in the training data. In our application, the optimal number of training images was determined to be close to 700. The spatial distribution of the selected training positions is depicted in Figure 15.

**Figure 16:**
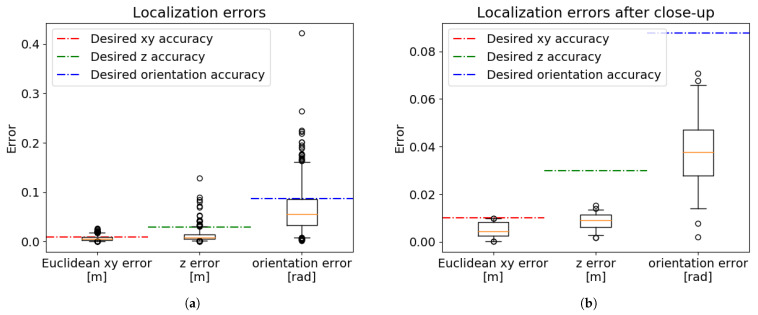
Results of the two-phase localization system. The results are presented using the five-number summary, with outliers (2%) depicted by circles. (**a**) All test data. (**b**) After iterative close-up.

Figure 16a shows the results of the trained neural networks in comparison to the ground-truth labels from the testing data. The results of the bin-picking process were negatively influenced by often having only a part of the manipulated object in the field of view of the camera. To increase the robustness of the system, the iterative close-up method was implemented (Section 2.1), in which we adjust the camera position after each captured image. Repeating this adjustment for three iterations allows the camera to get to a position with an ideal view of the object, further increasing the precision of the localization. Figure 16b shows the results of localization using the last image after the iterative close-up procedure.

Desired accuracy values depicted in Figure 16 were estimated after initial testing. The desired accuracy in the *x* and *y* axes was estimated by manually placing the suction cups in an optimal gripping position and shifting them in the horizontal direction. The desired orientation accuracy was estimated by a series of experiments on a part with a known position. We estimate the maximal orientation error to be 5° (0.087 rad). Arguably, there is no upper limit on the *z*-axis error, since the gripping motion is stopped at the moment of contact based on the force feedback. However, since the robot has to slow down during such movements, we have set the desired *z*-axis accuracy to a reasonable value. The desired accuracy values are shown in Equation (22). The precision of the final system was deemed suitable for use in the bin-picking application.

(22) $$\begin{array}{c} \sqrt{x_{er}^{2}+y_{er}^{2}}\;<0.01\;,\\ \left|z_{er}\right|\;<0.03\;,\\ \left|rx_{er}\right|+\left|ry_{er}\right|\;<0.087 \end{array}$$

Running the models that are based on neural networks does not require extensive computational resources. In comparison, the execution time of the HOG detector was considerably higher. To speed up the detector, the original 2592 × 1944 pixel input image was downscaled to 25% of the original resolution. Table 1 shows the computational time required for one feed-forward run and memories used for loading the models for the position and orientation CNN, occlusion CNN, and HOG object detector. The computational times were averaged from 1000 executions. The experiments were performed on a regular personal computer with an Intel Xeon(R) E3-1240 v5 @ 3.50 GHz processor (Intel Corporation, Santa Clara, CA, USA) and 32 GB RAM. No graphics card was employed, and the same computer was used for training the models.

It should be noted that the CNN networks are executed on every object detection, while HOG is executed only once per image. As described in Section 2.1, multiple objects are detected during the selection phase, and generally, only one object is detected during the iterative alignment. Therefore, the computational time varies slightly depending on the phase of the bin-picking process. Nevertheless, the HOG detector creates a bottleneck in the localization pipeline. However, the designed localization pipeline is modular and the detector can be replaced by a different method if necessary, as discussed in Section 3.1.

Since the models in this system are comparatively small, it is feasible to perform the training without using a GPU. Training the neural network model for pose estimation required approximately 13 min, while the HOG model training time was even lower.

Based on the results of the BOP challenge 2020 [26], the CosyPose method [20] had the best performance on the T-LESS (texture-less) dataset relevant to our problem. CosyPose allows for pose estimation from RGB images, however, it is unsuitable for use with our dataset due to its training data requirements. Thus, we will be providing only a comparison regarding the necessary training prerequisites and the training and inference complexity. Similar to the majority of the current state-of-the-art methods, CosyPose requires a 3D model of the object, as well as object masks and depth data for training. These are difficult to acquire, especially for reflective parts, for which a traditional structure from motion methods and even 3D cameras produce poor results. Additionally, the training complexity is much higher in comparison with our method. In the original paper [20], the method was trained using 32 GPUs and the training took a considerable amount of time. The computational time for the pose estimation phase stated in the BOP leaderboards is approximately 500 ms, in this regard, we outperformed CosyPose almost three times. We are also significantly faster than most of the other methods presented in the BOP challenge.

We provide all images used during the training and testing phase in a publicly available dataset. In this work, we use approximately 3000 images captured with the exposure time of 7.5 ms. However, to further enhance the data, all images were initially taken with four different exposure times, namely: 3.75, 7.5, 15, and 33 ms. This resulted in a dataset with close to 12,000 labeled images. The labeled images were later used in [36] for visual data simulation for deep learning, in which we explored the possibility of using synthetic data for the developed detection and localization system. The system trained on synthetic data was later successfully tested with comparable results to the original system trained on real images.

### 4.2. Factory Setup

**Figure 17:**
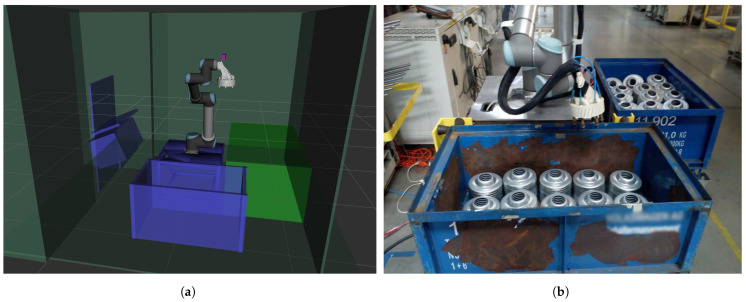
Robotic work cell. (**a**) The model of the robotic workspace created in MoveIt libraries. (**b**) The setup of the robotic cell in the factory.

The laboratory system was modified for use in a real factory environment. A complete robotic cell was designed, integrating industrial-grade components into the original solution. A model of the workspace (Figure 17a) was implemented using MoveIt libraries [37] from the ROS system [38]. The MoveIt libraries were then used to plan trajectories for the robot. The regular personal computer used during laboratory testing was replaced with an industrial-grade computer equipped with an Intel(R) Xeon(R) CPU E3-1268L v3 2.30 GHz processor and 8 GB RAM. Safety elements, a system logger, and a graphical user interface with basic control options were added. The complete system setup can be seen in Figure 17b. Since the production was running during the full day and night cycle, there was a large difference in lighting conditions compared to the laboratory environment. The system was retrained on-site to compensate for these changes, creating the aforementioned labeled image dataset.

The system was deployed in a welding hall polluted with a constant presence of swarf and dust particles, therefore, additional shielding had to be added to the camera and other vulnerable electrical equipment. The original gripper prototype was redesigned and topologically optimized. The prolonged continuous use of the camera resulted in overheating and frequent malfunctions in the image acquisition pipeline, and additional cooling was installed in the form of an external ventilator.

To ensure robust and fail-safe control, the system was extended to contain a finite-state automaton controlling the full bin-picking process. The process starts with the robot taking images of the whole transportation container and selecting several reachable parts. The robot then picks individual parts from the selection and places them in the assembly line. A set of recovery measures was embedded in the system. In case of a failure during the process, the system either automatically restarts the picking process or signalizes the failure to the operator, together with necessary actions. Although the application is designed to be autonomous, multiple situations cannot be resolved using the developed system. Parts flipped upside down are not considered for manipulation and should be removed manually prior to the process. The geometry of the gripper does not allow to reach parts that are inclined towards the wall of the transportation container, and in these situations, the robot signalizes that the part is unreachable and continues in the process by selecting another one.

### 4.3. Results of Long-Term Testing

The system was connected to the automated assembly line for several weeks without any supervision or interference, only controlled by local operators. In the final testing phase, the bin-picking application ran for 175 h, including the time spent waiting for the assembly line or the operator. The bin-picking process itself ran for 56 full hours. Out of the 3469 parts picked during the final testing phase, the application performed with a success rate of 98.5%, and the remaining 1.5% required actions from the operator.

The required tact time for the bin-picking process set by the customer was 60 s per one part. During the final testing phase, our system achieved an average tact time of 58.4 s with a median of 45.3 s. Since the robot was operating in collaborative mode, its maximal speed was limited, and 78.2% of the overall picking time was taken by the movement of the robot. The remaining time-consuming processes were: motion planning 7%, lighting and image acquisition 5.2%, and image processing and pose calculation 2.5%. To further reduce the picking time, we could either switch the robot to operate in a non-collaborative mode, or use a different type of robotic manipulator.

## 5. Conclusions

The proposed bin-picking system diverts from traditional pose estimation methods by its focus on deployment in industrial applications. While the use of neural networks and machine learning in general often requires specific and expensive hardware, the pose detection pipeline presented in this paper is designed with its applicability in mind. The developed system supports different hardware for training and running the models and can be used on any reasonably equipped computer, making it a viable low-cost solution for industrial use. The proposed vision system also allows for the use of a cheap 2D camera.

We present a modular two-phase, data-driven detection and localization method for 6DoF object pose estimation. We also introduce a new representation of object position and orientation that is suitable for training an end-to-end neural network without the need for a 3D model or a hard-coded geometry of the object. The localization system is further extended by an occlusion detection module.

The object considered in this work is a relatively simple rotationally symmetric part. However, the difficulty of the localization task increases because of the reflective properties of the part. The appearance of industrial metallic parts is highly dependant on their orientation, illumination, and the viewpoint of the camera. We proposed a vision system that uses a high-intensity light source in a fixed position to a 2D camera mounted directly on a robotic gripper. This allowed us to take advantage of the reflective properties of metallic objects for pose estimation. Furthermore, using this setup, a semi-automatic data collection process was devised. This provides a useful tool for labeled image acquisition and allows for data acquisition on-site. The localization method estimates the camera to the object position and is independent of the robotic manipulator. This allows for an easy transfer between different robotic solutions. Provided that the camera and lighting systems remain unchanged, the pose estimation method can be transferred without retraining. Additionally, our previous work proved that the system can also be used with synthetic training data [36].

A complete robotic cell was designed and the experimental laboratory setup was modified for use in an industrial environment. The system was extended to contain a finite-state automaton with a set of recovery measures and a basic control interface for the operator, to provide fully automatic and fail-safe robot control without the need for any supervision or interference. The system was successfully deployed and extensively tested in factory production.

In future work, we hope to test our framework on a more complex and rotationally non-symmetric object. This would require modifying the orientation model by adding the rotation around the object normal. However, since the proposed system does not rely on hard-coded properties of the object, using this framework on an object whose geometry is more complex should not bring additional difficulties, as the neural network model will learn the necessary visual features.

## Figures and Tables

**Table 1:** Requirements on individual neural networks.

	Computational Time	Model Size
Position and orientation CNN	1.98 ms	50.8 MB
Occlusion CNN	1.92 ms	50.8 MB
HOG	120.28 ms	29.6 kB

## Data Availability

The data presented in this study are openly available at: http://imr.ciirc.cvut.cz/Downloads/Datasets (accessed on 9 September 2021).

## References

[1] Correll N., Bekris K., Berenson D., Brock O., Causo A., Hauser K., Okada K., Rodríguez A., Romano J., Wurman P. (2016). Analysis and Observations From the First Amazon Picking Challenge. IEEE Trans. Autom. Sci. Eng..

[2] Martínez C., Boca R., Zhang B., Chen H., Nidamarthi S. Automated bin-picking system for randomly located industrial parts. Proceedings of the 2015 IEEE International Conference on Technologies for Practical Robot Applications (TePRA).

[3] Rodrigues J.J., Kim J.S., Furukawa M., Xavier J., Aguiar P., Kanade T. 6D pose estimation of textureless shiny objects using random ferns for bin-picking. Proceedings of the 2012 IEEE/RSJ International Conference on Intelligent Robots and Systems.

[4] Shen X. (2019). A survey of Object Classification and Detection based on 2D/3D data. arXiv.

[5] Xu H., Xu J., Xu W. (2019). Survey of 3D modeling using depth cameras. Virtual Real. Intell. Hardw..

[6] Lowe D.G. (2004). Distinctive Image Features from Scale-Invariant Keypoints. Int. J. Comput. Vis..

[7] Tola E., Lepetit V., Fua P. A fast local descriptor for dense matching. Proceedings of the 2008 IEEE Conference on Computer Vision and Pattern Recognition.

[8] Park K., Patten T., Vincze M. Pix2pose: Pixel-wise coordinate regression of objects for 6d pose estimation. Proceedings of the IEEE International Conference on Computer Vision.

[9] Ulrich M., Wiedemann C., Steger C. CAD-based recognition of 3D objects in monocular images. Proceedings of the 2009 IEEE International Conference on Robotics and Automation.

[10] Hinterstoisser S., Lepetit V., Ilic S., Holzer S., Bradski G., Konolige K., Navab N. (2012). Model based training, detection and pose estimation of texture-less 3d objects in heavily cluttered scenes. Asian Conference on Computer Vision.

[11] Rodrigues J.J.M. (2018). 3D Pose Estimation for Bin-Picking: A Data-Driven Approach Using Multi-Light Images. Ph.D. Thesis.

[12] Zhao Z.Q., Zheng P., Xu S.T., Wu X. (2019). Object Detection With Deep Learning: A Review. IEEE Trans. Neural Netw. Learn. Syst..

[13] Rad M., Lepetit V. BB8: A Scalable, Accurate, Robust to Partial Occlusion Method for Predicting the 3D Poses of Challenging Objects without Using Depth. Proceedings of the IEEE International Conference on Computer Vision.

[14] Zhao Z., Peng G., Wang H., Fang H.S., Li C., Lu C. (2018). Estimating 6D Pose From Localizing Designated Surface Keypoints. arXiv.

[15] Hu Y., Hugonot J., Fua P., Salzmann M. Segmentation-Driven 6D Object Pose Estimation. Proceedings of the IEEE Conference on Computer Vision and Pattern Recognition.

[16] Peng S., Liu Y., Huang Q., Zhou X., Bao H. PVNet: Pixel-Wise Voting Network for 6DoF Pose Estimation. Proceedings of the 2019 IEEE/CVF Conference on Computer Vision and Pattern Recognition (CVPR).

[17] Xiang Y., Schmidt T., Narayanan V., Fox D. PoseCNN: A Convolutional Neural Network for 6D Object Pose Estimation in Cluttered Scenes. Proceedings of the Robotics: Science and Systems 2018.

[18] Tekin B., Sinha S., Fua P. Real-Time Seamless Single Shot 6D Object Pose Prediction. Proceedings of the IEEE Conference on Computer Vision and Pattern Recognition.

[19] Kehl W., Manhardt F., Tombari F., Ilic S., Navab N. SSD-6D: Making RGB-Based 3D Detection and 6D Pose Estimation Great Again. Proceedings of the 2017 IEEE International Conference on Computer Vision (ICCV).

[20] Labbe Y., Carpentier J., Aubry M., Sivic J. CosyPose: Consistent multi-view multi-object 6D pose estimation. Proceedings of the European Conference on Computer Vision (ECCV).

[21] Pinto L., Gupta A. Supersizing self-supervision: Learning to grasp from 50k tries and 700 robot hours. Proceedings of the 2016 IEEE International Conference on Robotics and Automation (ICRA).

[22] Levine S., Pastor P., Krizhevsky A., Quillen D. (2016). Learning Hand-Eye Coordination for Robotic Grasping with Deep Learning and Large-Scale Data Collection. Int. J. Robot. Res..

[23] Calli B., Singh A., Walsman A., Srinivasa S., Abbeel P., Dollar A. The YCB object and Model set: Towards common benchmarks for manipulation research. Proceedings of the 2015 International Conference on Advanced Robotics (ICAR).

[24] Krull A., Brachmann E., Michel F., Yang M.Y., Gumhold S., Rother C. Learning Analysis-by-Synthesis for 6D Pose Estimation in RGB-D Images. Proceedings of the IEEE International Conference on Computer Vision (ICCV).

[25] Hodaň T., Michel F., Brachmann E., Kehl W., Glent Buch A., Kraft D., Drost B., Vidal J., Ihrke S., Zabulis X. BOP: Benchmark for 6D Object Pose Estimation. Proceedings of the European Conference on Computer Vision (ECCV).

[26] Hodaň T., Sundermeyer M., Drost B., Labbé Y., Brachmann E., Michel F., Rother C., Matas J. BOP Challenge 2020 on 6D Object Localization. Proceedings of the European Conference on Computer Vision Workshops (ECCVW).

[27] Hodaň T., Haluza P., Obdržálek S., Matas J., Lourakis M., Zabulis X. T-LESS: An RGB-D Dataset for 6D Pose Estimation of Texture-Less Objects. Proceedings of the 2017 IEEE Winter Conference on Applications of Computer Vision (WACV).

[28] Dalal N., Triggs B. Histograms of oriented gradients for human detection. Proceedings of the 2005 IEEE Computer Society Conference on Computer Vision and Pattern Recognition.

[29] Walsh J., O’ Mahony N., Campbell S., Carvalho A., Krpalkova L., Velasco-Hernandez G., Harapanahalli S., Riordan D. (2019). Deep Learning vs. Traditional Computer Vision. Science and Information Conference.

[30] Jiao L., Zhang F., Liu F., Yang S., Li L., Feng Z., Qu R. (2019). A Survey of Deep Learning-Based Object Detection. IEEE Access.

[31] Redmon J., Divvala S., Girshick R., Farhadi A. You Only Look Once: Unified, Real-Time Object Detection. Proceedings of the IEEE Conference on Computer Vision and Pattern Recognition 2016.

[32] Ren S., He K., Girshick R., Sun J. (2015). Faster R-CNN: Towards Real-Time Object Detection with Region Proposal Networks. IEEE Trans. Pattern Anal. Mach. Intell..

[33] Braverman V., Kao M.Y. (2016). Sliding Window Algorithms. Encyclopedia of Algorithms.

[34] Cortes C., Vapnik V. (1995). Support-vector networks. Mach. Learn..

[35] Abadi M., Barham P., Chen J., Chen Z., Davis A., Dean J., Devin M., Ghemawat S., Irving G., Isard M. (2016). TensorFlow: A System for Large-Scale Machine Learning. Proceedings of the 12th USENIX Conference on Operating Systems Design and Implementation (OSDI’16).

[36] Surák M., Košnar K., Kulich M., Kozák V., Přeučil L. (2019). Visual Data Simulation for Deep Learning in Robot Manipulation Tasks. International Conference on Modelling and Simulation for Autonomous Systems.

[37] Coleman D., Sucan I., Chitta S., Correll N. (2014). Reducing the Barrier to Entry of Complex Robotic Software: A MoveIt! Case Study. arXiv.

[38] Quigley M., Conley K., Gerkey B., Faust J., Foote T., Leibs J., Wheeler R., Ng A. (2009). ROS: An open-source Robot Operating System. ICRA Workshop Open Source Softw..

